# The Midwives Bill

**Published:** 1918-02-09

**Authors:** 


### NURSING AFFAIRS IN IRELAND.

### The Midwives Bill.

The Midwives Bill has now passed through the House of Commons, and before this issue of The Hospital is in the hands of its readers will probably have become law in its present amended form. It will not come into operation until January 1 of next year, although in its original form it was designed to have become operative a year earlier. This is owing to the fact that the Bill was drawn and presented to the Government by the Local Government Board in 1916, but no suitable opportunity of submitting it to Parliament occurred until last November. After it has been placed on the Statute Book a great deal of time must elapse before the necessary machinery can be set up.

The board is to consist of eleven members, two of whom are to be appointed after consultation with county and county borough councils, which will be the supervising authority under the Act. Four members of the board are to be elected by the registered medical practitioners of Ireland, and four midwives' representatives are to be appointed after consultation with recognised nursing associations in Ireland. Such a board is bound to be an efficient body thoroughly acceptable to all parties concerned, but its formation will be unavoidably slow, and the current year will be well on its way before it is complete.

Financial Provisions.

As soon as the Irish Midwives Board is appointed its first duty will be to draw up a code of regulations governing future conditions of training and registration, as well as specifying and defining the duties of midwives, the limits of their practice, and providing against malpractice, negligence, or misconduct. A secretary, who will have the custody, preparation, and correction of the Roll of Midwives, will have to be appointed. This person may be a man or a woman, whose appointment and salary must be approved by the Privy Council. None of the members of the board will be paid salaries, but will be allowed reasonable expenses for attendance. These, together with the secretary's salary and all other charges incurred, will be payable out of such funds as the board may derive from registration fees, and, if there is any shortage, the difference will be levied on the county and county boroughs and charged against the rates. The duty of inspecting will fall on the supervising authority, and not on the board.

Although the Act will come into operation on January 1, 1919, the unqualified and unregistered midwife will not be automatically guillotined, so long as she makes no pretence that she is otherwise. The immediate effect of the new law will therefore be to separate the sheep from the goats, and to brand them. The unqualified and unregistered may continue to ply their trade until January 1, 1920, a period of grace which is not unreasonable.

An excellent provision of the Bill ensures reciprocity of employment in His Majesty's Dominions for qualified Irish midwives who may desire to migrate. At present no Irish qualification in midwifery, however high, entitles the holder to practise in England without first passing the English C.M.B. examination, whereas an English midwife may practise with impunity in Ireland. It is now provided that a properly certified and registered midwife coming from any part of His Majesty's Dominions may practise in Ireland on condition that the country from whence she comes adopts a reciprocal attitude. Of course, this Irish disability arose from the absence of any midwifery legislation in this country, and, so far as England is concerned, I understand that the C.M.B. is altogether sympathetic to the matter being adjusted in the manner named.

The Irish Local Government Board, and especially the Medical Commissioner, Doctor Coey Bigger, is warmly to be congratulated on having successfully engineered this urgently needed reform through Parliament at a most difficult time. A great many objections and amendments were lodged by individuals and societies who failed to recognise the risk of wrecking the measure by making it contentious. By diplomatic handling and earnest endeavour Dr. Bigger has succeeded in overcoming these difficulties, and making it generally acceptable. This is by a long way the most forward and practical step that has yet been taken in this country in the cause of child welfare.

